# A Phase II Randomized Clinical Trial and Mechanistic Studies Using Improved Probiotics to Prevent Oral Mucositis Induced by Concurrent Radiotherapy and Chemotherapy in Nasopharyngeal Carcinoma

**DOI:** 10.3389/fimmu.2021.618150

**Published:** 2021-03-24

**Authors:** Chaofei Xia, Chunling Jiang, Wenyu Li, Jing Wei, Hu Hong, Jingao Li, Liu Feng, Hong Wei, Hongbo Xin, Tingtao Chen

**Affiliations:** ^1^National Engineering Research Center for Bioengineering Drugs and Technologies, Institute of Translational Medicine, Nanchang University, Nanchang, China; ^2^Department of Radiation Oncology, Jiangxi Cancer Hospital, Nanchang, China; ^3^NHC Key Laboratory of Personalized Diagnosis and Treatment of Nasopharyngeal Carcinoma (Jiangxi Cancer Hospital of Nanchang University), Nanchang, China; ^4^Precision Medicine Institute, The First Affiliated Hospital, Sun Yat-sen University, Guangzhou, China

**Keywords:** probiotics, oral mucositis, intestinal microbiota, nasopharyngeal cancer, radiotherapy and chemotherapy

## Abstract

Earlier evidence has proven that probiotic supplements can reduce concurrent chemoradiotherapy (CCRT)-induced oral mucositis (OM) in nasopharyngeal cancer (NPC). The incidence of severe OM (grade 3 or higher) was the primary endpoint in this study. We first enrolled 85 patients with locally advanced NPC who were undergoing CCRT. Of them, 77 patients were finally selected and randomized (1:1) to receive either a probiotic cocktail or placebo. To investigate the protective effects and the mechanism of probiotic cocktail treatment on OM induced by radiotherapy and chemotherapy, we randomly divided the rats into the control (C) group, the model (M) group, and the probiotic (P) group. After treatment, samples from the tongue, blood, and fecal and proximal colon tissues on various days (7th, 14th, and 21st days) were collected and tested for the inflammatory response, cell apoptosis, intestinal permeability, and intestinal microbial changes. We found that patients taking the probiotic cocktail showed significantly lower OM. The values of the incidence of 0, 1, 2, 3, and 4 grades of OM in the placebo group and in the probiotic cocktail group were reported to be 0, 14.7, 38.2, 32.4, and 14.7% and 13.9, 36.1, 25, 22.2, and 2.8%, respectively. Furthermore, patients in the probiotic cocktail group showed a decrease in the reduction rate of CD3^+^ T cells (75.5% vs. 81%, *p* < 0.01), CD4^+^ T cells (64.53% vs. 79.53%, *p* < 0.01), and CD8^+^ T cells (75.59 vs. 62.36%, *p* < 0.01) compared to the placebo group. In the rat model, the probiotic cocktail could ameliorate the severity of OM, decrease the inflammatory response, cause cell apoptosis and intestinal permeability, and restore the structure of gut microbiota to normalcy. In conclusion, the modified probiotic cocktail significantly reduces the severity of OM by enhancing the immune response of patients with NPC and modifying the structure of gut microbiota.

**Clinical Trial Registration:** The Clinical Trial Registration should be the NCT03112837.

## Introduction

Nasopharyngeal carcinoma (NPC) is a prevalent malignant neoplasm in southern China, and concurrent chemoradiotherapy (CCRT) is the standard treatment for locally advanced NPC worldwide (1). Toxic side effects caused by CCRT occur during and after the treatment, and oral mucositis (OM) is probably the most common complication in patients with head-and-neck cancer (accounting for ~80%) (2). OM not only interferes with the quality of the life of the patient but also gives rise to a 19% interruption rate in radiotherapy or CCRT (3). Though topical agents, such as palifermin, chlorhexidine, actovegin, kangfuxin, royal jelly, zinc supplement, benzydamine, cryotherapy, laser therapy, and professional oral hygiene, are used for CCRT-induced OM, there is still no accepted standard therapy for the prevention and treatment of OM (4). Therefore, a feasible and effective method to prevent OM during cancer treatment is urgently required.

The intestinal microbiota has become an important regulator of host immunity and may affect the outcome of cancer immunotherapy (5–7). Cancer treatments, such as radiotherapy and chemotherapy, might cause a decrease in the immunity of patients with cancer, eventually aggravating immunotherapy-induced mucosal toxicity (8). Evidence from studies on humans and experimental animals suggested that intestinal microbiota, such as probiotics, could modulate the anti-cancer immune response and attenuate cancer treatment-related toxic side effects (9–11). Oral supplementation of *Bifidobacterium*, alone or with anti-programed cell death of protein 1 ligand 1 (PDL1), in mice had promoted CD8^+^T cell-induced anti-tumor immunity (12). In line with this study, Vetizou et al. (10) also found that *Bacteroidales* played an important role in the immunostimulatory effects of cytotoxic T lymphocyte-associated antigen 4 (CTLA-4) blockade by promoting the maturation of intratumoral dendritic cells and with a T_H_1 response detected in the lymph nodes of the draining tumor.

In a previous study, the probiotic drugs, such as *Bifidobacterium longum, Lactobacillus lactis and Enterococcus faecium*, exerted a therapeutic effect and could reduce the severity of OM in patients with NPC, who underwent CCRT, and greatly increased the number of immune cells (13). However, all of the probiotic drugs in China were approved 10–20 years ago, and some problems, such as the misidentified bacteria on the label or the use of potential pathogens, such as *E. faecium* and *Bacillus cereus*, in drugs, hindered their further development. For example, *E. faecium* has been considered as a probiotic and is used in more than 75% of probiotic drugs, while the latter strain is considered as an opportunistic pathogen other than as a probiotic due to its multi-drug resistance and virulent factors (14).

Therefore, in the present study, we first isolated *Lactobacillus plantarum* from the feces of a healthy crowd living in the cancer-free village through high-throughput sequencing analysis. Then, the above *L. plantarum* and *Bifidobacterium animalis*, which were isolated from Bama Changshou Village 30 years ago, were mixed with *Lactobacillus rhamnosus* and *Lactobacillus acidophilus*to form a probiotic cocktail. Next, the effectiveness of the probiotic cocktail on OM in patients with NPC was investigated *via* the randomized, double-blind, placebo-controlled trial. Finally, the possible protective mechanism of the probiotic cocktail on OM was further investigated through the OM rat model.

## Materials and Methods

### Screening Bacteria From Cancer-Free Village Residents

Fecal samples from healthy people (HP) (*n* = 5, healthy employees from the Jiangxi Cancer Hospital), tumor patients (TP) (*n* = 5, tumor patients from the Jiangxi Cancer Hospital), and non-cancer people (NT) (*n* = 5, healthy residents from the cancer-free village, Wuyuan, Jiangxi, Nanchang, PR, China) were collected in June 2016, and high-throughput sequencing analysis was used to compare the microbial diversity among these samples. A viable counting method was used to isolate bacteria with the de Man–Rogosa–Sharpe (MRS) medium by mainly screening for *Lactobacillus* spp. from the feces of cancer-free village residents, and the isolates were identified using a gene sequencing technology (15).

### Evaluation of the Probiotic Characteristics of the Selected Probiotics *in vitro*

The *L. plantarum* MH-301 was isolated from cancer-free village residents, and *B. animalis* subsp. *Lactis* LPL-RH (Harbin Meihua Biotechnology Co., Ltd., Harbin, Heilongjiang, PR China and Isolated from Changshou Village, Bama Town in Guangxi Province, China), *L. rhamnosus* LGG-18 (Harbin Meihua Biotechnology Co., Ltd., Harbin, Heilongjiang, PR China), and *L. acidophilus* (Harbin Meihua Biotechnology Co., Ltd., Harbin, Heilongjiang, PR China) were selected to make a probiotic cocktail, and the acid tolerance test (16), the anti-oxidative test (17), the antimicrobial test (18), the adherence assay test (19), and the adherence assay test (19) were conducted to evaluate the probiotic characteristics of the selected strains.

### Evaluation of the Probiotic Cocktail on Reducing the Side Effects Induced by CCRT in Patients With NPC

Male and female patients (18–70 years old) diagnosed with locally advanced NPC were enrolled for the randomized, double-blind, placebo-controlled trial at the Jiangxi Cancer Hospital in China. The clinical stage of the patient was determined according to the 8th edition of the International Union Against Cancer/American Joint Committee on Cancer TNM staging system, and patients diagnosed with NPC without distant metastasis and who had a Karnofsky score were enrolled. Patients with a previous history of cancer or co-existing tumors, who were unable to take oral medicine and/or absorb drugs in the digestive tract, who had a high risk for antimicrobial agents, who had OM or recurrent OM before CCRT, and who had serious and/or uncontrollable infections, or other diseases were excluded.

We first enrolled 85 patients with locally advanced NPC who were undergoing CCRT. About 77 patients were finally selected and randomized (1:1) to receive either a probiotic mixture or a placebo. The process of random allocation is as follows: the random allocation sequence was performed at a ratio of 1:1 using nQueryAdvisor®v7.0 software, which uses a pseudo-random number generator. The randomization sequence was produced before the first enrollment. The executor performed inclusion following the inclusion and exclusion criteria. Then, patients with NPC were assigned to the probiotic cocktail group or the control group by the clinical research technician who was also blinded. According to the double-blind method, those who participated in this study did not know the type of treatment that each patient with NPC received.

According to the guidelines of the National Comprehensive Cancer Network (NCCN), all patients underwent cisplatin chemotherapy [32 fractions of 70 Gy radiotherapy (2.19 Gy/d, 5 d/wk) with a total tumor volume and a clinical target volume of 60 Gy] and intensity-modulated radiation therapy (IMRT) [32 fractions for 45 days (6–7 weeks in total) and intravenously infused with cisplatin (100 mg/m^2^) on days 1, 22, and 43].

Oral probiotic cocktail (containing *L. plantarum* MH-30110^9^ CFU, *B. animalis* subsp. *Lactis* LPL-RH10^9^ CFU, *L. rhamnosus* LGG-1810^9^ CFU, and *L. acidophilus* 10^9^ CFU), or placebo were supplied to patients for 7 weeks (one capsule, 2 times a day) from the first day of chemoradiotherapy to the end. The severity, occurrences, and symptoms of OM were evaluated by at least two advanced radiation oncologists [the common terminology of National Cancer Institute for adverse events (version 4.0)]. The short-term efficacy response rates (the Response Evaluation Criteria in Solid Tumors based on MRI) were evaluated for patients with complete and partial radiotherapy responses after the completion of CCRT (20). Patient weights were recorded weekly; their biochemical parameter analysis, determination of lymphocyte immunity, and routine blood analysis were measured.

This study was approved by the local clinical research Ethics Committee and was conducted by following the Declaration of Helsinki (Clinical Trials number, NCT03112837). All patients gave their informed consent before the trial.

### Total Bacterial Genomic DNA Extraction and High-Throughput Sequencing of Human Fecal Bacteria

Microbial DNA was obtained from the fecal samples of the HP group (from the employees of Jiangxi Cancer Hospital, *n* = 10), before the treatment of radiotherapy plus chemotherapy plus a placebo (BRCP) group (*n* = 10), before the treatment of radiotherapy plus chemotherapy plus the probiotic combination (BRCPM) group (*n* = 10), after treatment with radiotherapy plus chemotherapy plus a placebo (ARCP) group (*n* = 10), and after treatment with radiotherapy plus chemotherapy plus the probiotic combination ARCPM group (*n* = 10). Samples were kept at −80°C until DNA extraction. Bacterial genomic DNA was extracted from fecal samples using the DNA magnetics and extract kit (Tiangen Biotech, Beijing, China), according to the instruction of the manufacturer. Total genomic DNA was amplified with a forward primer, F341 5′-ACT CCT ACG GGR SGC AGC AG-3′, and a reverse primer, R806 5′- GGA CTA CVV GGG TAT CTA ATC-3′ that amplified the regions from V3 to V4 of the 16S ribosomal DNA gene for high-throughput sequencing analysis (PRJNA579226) (21).

### Development of OM Rat Model and Treatment

Male Sprague-Dawley rats, aging 8–10 weeks, were purchased from Hunan Si Lake King of Experimental Animal Co., Ltd. (Changsha, Hunan, China). The rats were habituated to the animal facility for 2 weeks before beginning the experiment and kept under a 12 h light/dark cycle, with a temperature of 21 ± 1°C and humidity of 55 ± 10%. Food and water were given *ad libitum*. Animal care and procedures were followed under the guidelines of the National Institutes of Health and the Care and Use of Laboratory Animals. All experiments were approved by the Ethical Committee of the Nanchang University.

For the mucositis model, both radiation and chemotherapy were used for the induction of OM with busulfan (Sigma-Aldrich, MO, USA) at a dose of 6 mg/kg for 4 days of chemotherapy (22). The rats were anesthetized with an intraperitoneal injection of ketamine (Rotex, Trittau, Germany) before irradiation. Then, the rats were irradiated one at a time in the head region with 20 Gy, using Clinac 600C, a 4-MV therapeutic linear accelerator (Varian Medical Systems Inc., Palo Alto, CA, USA) at a dose rate of 2 Gy/min, to expose the oral mucosa to radiation, where a 1.5 cm bolus was used for the radiation dose buildup (23). The rats were divided into three groups: the control (C) group (*n* = 13) received an identical volume of gelatine physiological saline (i.g.) for 21 days without radiotherapy and chemotherapy; the model (M) group (*n* = 13) was treated with an identical volume of i.g. for 21 days with radiotherapy and chemotherapy; and the probiotic (T) group (*n* = 13) was pretreated with a probiotic combination containing *L. plantarum* MH-30110^9^ CFU, *B. animalis* subsp. *Lactis* LPL-RH10^9^ CFU, *L. rhamnosus*LGG-1810^9^ CFU, and *L. acidophilus* 10^9^ CFU, for 7 days (1 ml, 1 time a day) before radiotherapy and chemotherapy. All animals were monitored daily to examine the status of the oral cavity, the amount of oral intake, weight, and survival.

### Histopathological Examination

To evaluate the histopathologic changes in the tongue, five rats in each group were sacrificed on the 7th and 14th days, and three rats in each group were sacrificed on the 21st day. The mucosa from the tongue was collected at the end of the experiment. The tongue was exposed and photographed, tongue samples (collected on 7th, 14th, and the 21st days) were divided into two parts separately, and one part was fixed in 10% buffered formalin for 48 h and embedded in paraffin. Then, multiple sections (4 μm thick) were deparaffinized with xylene and stained with H&E. The remaining part of the tongue samples was used for quantitative real-time PCR (qRT-PCR) and Western blot. Samples of blood and feces were obtained for biochemical assays and high-throughput sequencing (PRJNA579226), respectively.

### RNA Extraction and qRT-PCR Analysis

Total RNA was extracted from the tongue tissue using the Tri Reagent Kit (Sigma Aldrich, MO, USA) according to the instructions of the manufacturer. Equal amounts of RNA were used to synthesize complementary DNA (cDNA) using the Fast Quant RT Kit (Tiangen, Beijing, China). The cDNA was used for qRT-PCR with the KAPA SYBR FAST Universal 2× qPCR Master Mix (Kapa Biosystems, MA, USA). The expression of IL-1β, IL-6, tumor necrosis factor α (TNF-α), and the housekeeping gene, such as glyceraldehyde 3-phosphate dehydrogenase (GAPDH), was assessed by qRT-PCR. The relative amount of transcripts for target genes was determined for each cDNA sample after normalization against GAPDH. The data were analyzed using the 2^−ΔΔCT^ method. The following primers were used: GAPDH, 5′-AGC CAA AAG GGT CAT CAT CT-3′ (forward) and 5′-GGG GCC ATC CAC AGT CTT CT-3′ (reverse); IL-6, 5′-GAA ATC GTG GAA ATG AG-3′ (forward) and 5′-GCT TAG GCA TAA CGC ACT-3′ (reverse); IL-1β, 5′-GTG TCT TTC CCG TGG ACC TTC-3′ (forward) and 5′-TCA TCT CGG AGC CTG TAG TGC-3′ (reverse); TNF-α, 5′-GTG GAA CTG GCA GAA GAG GCA-3′ (forward) and 5′-AGA GGG AGG CCA TTT GGG AAC-3′ (reverse).

### Western Blot Analysis

The protein from the tongue and colon tissues was prepared with the RIPA lysis buffer containing protease and phosphatase inhibitors. Protein (25–30 μg) was loaded on 12% sodium dodecyl sulfate–polyacrylamide gel electrophoresis (SDS-PAGE) and transferred onto a polyvinylidene fluoride (PVDF) membrane. After blocking with 5% bovine serum albumin (BSA) (in TBS-T buffer), the membrane was incubated with a primary antibody, followed by incubation with the horseradish peroxidase (HRP)-conjugated IgG (1:5000, CST, USA). Protein bands were detected by an electro chemical luminescence (ECL) reagent and analyzed by the Flurochem System (FluorChemE, Cell Biosciences Inc., CA, USA). The primary antibody includes rabbit anti-GAPDH (1:5000, CST, Cat#5174), rabbit anti-NF-κB (1:1000, CST, Cat# 8242S), rabbit anti-phosphorylated-NF-κB (p-NF-κB; 1:1000, Abcam, Cat# ab86299), mouse anti-toll-like receptor 4 (TLR4; 1:1000, Santa Cruz, Cat# sc-293072), rabbit anti-B-cell lymphoma-2 (BCl-2; 1:1000, CST, Cat# 3498S), rabbit anti-BCl-2-associated-x-protein (Bax; 1:1000, CST, Cat# 14796S), rabbit anti-Claudin-1(1:1000, CST, Cat# 4933S), and rabbit anti-zonula occludens-1 (ZO-1; 1:1000, Cat# 5406S).

### Total Bacterial Genomic DNA Extraction and High-Throughput Sequencing of Animal Fecal Bacteria

Deoxyribonucleic acid extraction and high-throughput sequencing of animal fecal bacteria were consistent with the above human experiments. The fecal samples from C group, M group, and the T group were collected before sacrifice and kept at −80°C until DNA extraction. The DNA magnetics and extract kit (Tiangen, Biotech, Beijing, China) was used to extract the fecal bacterial genomic DNA, according to the instruction of the manufacturer. The total genomic DNA was amplified with the forward primer, F341 5′-ACT CCT ACG GGR SGC AGC AG-3′, and the reverse primer, R806 5′- GGA CTA CVV GGG TAT CTA ATC-3′ that amplified the regions from V3 to V4 of the 16S ribosomal DNA gene for high-throughput sequencing analysis (PRJNA579226).

### Data Analysis

The reported incidence of severe OM after receiving chemoradiotherapy in NPC is at 70–80% (24). Assuming that an average incidence of OM in the placebo and probiotics groups was at 74% and 34%, respectively, 70 patients were enrolled to ensure statistical significance (two-sided α = 0.05, 1–β = 0.9 and 1:1 ratio). We analyzed data from all randomized patients who received at least one dose of the drug. The most recent observations were used to estimate the missing values of the primary efficacy point, while the primary comparable analysis, secondary efficacy points, and the missing values for safety were not retained, and those values were analyzed to obtain the actual data.

Paired-end reads from the original DNA fragments were joined using FLASH Software (25). The joined pairs were quality filtered with the UPARSE software package, and the UPARSE pipeline was used to cluster the remaining sequences into operational taxonomic units (OTUs) at a minimum pair-wise identity of 97% (26). The annotated taxonomic information for each representative sequence, selected from each OTU, was determined using the ribosomal database project (RDP) classifier (27). The data from the OTUs were then used to calculate the Alpha-diversity (α-diversity) metrics by using QIIME (28). Distances between microbial communities obtained from different samples were calculated with the weighted UniFrac beta-diversity metric *via* QIIME (29). Non-metric multidimensional scaling (NMDS) analysis and principal coordinate analysis (PCoA) were used to visualize the pairwise UniFrac distances among the samples.

All data were reported as means and SD, and the results were analyzed with SPSS 23.0 software (SPSS, Inc., Chicago, Illinois) by the Student's *t*-test and the one-way ANOVA. The *p* < 0.05 was regarded as statistically significant.

## Results

### Screening of Probiotics From Cancer-Free Village Residents

**Figure 1:**
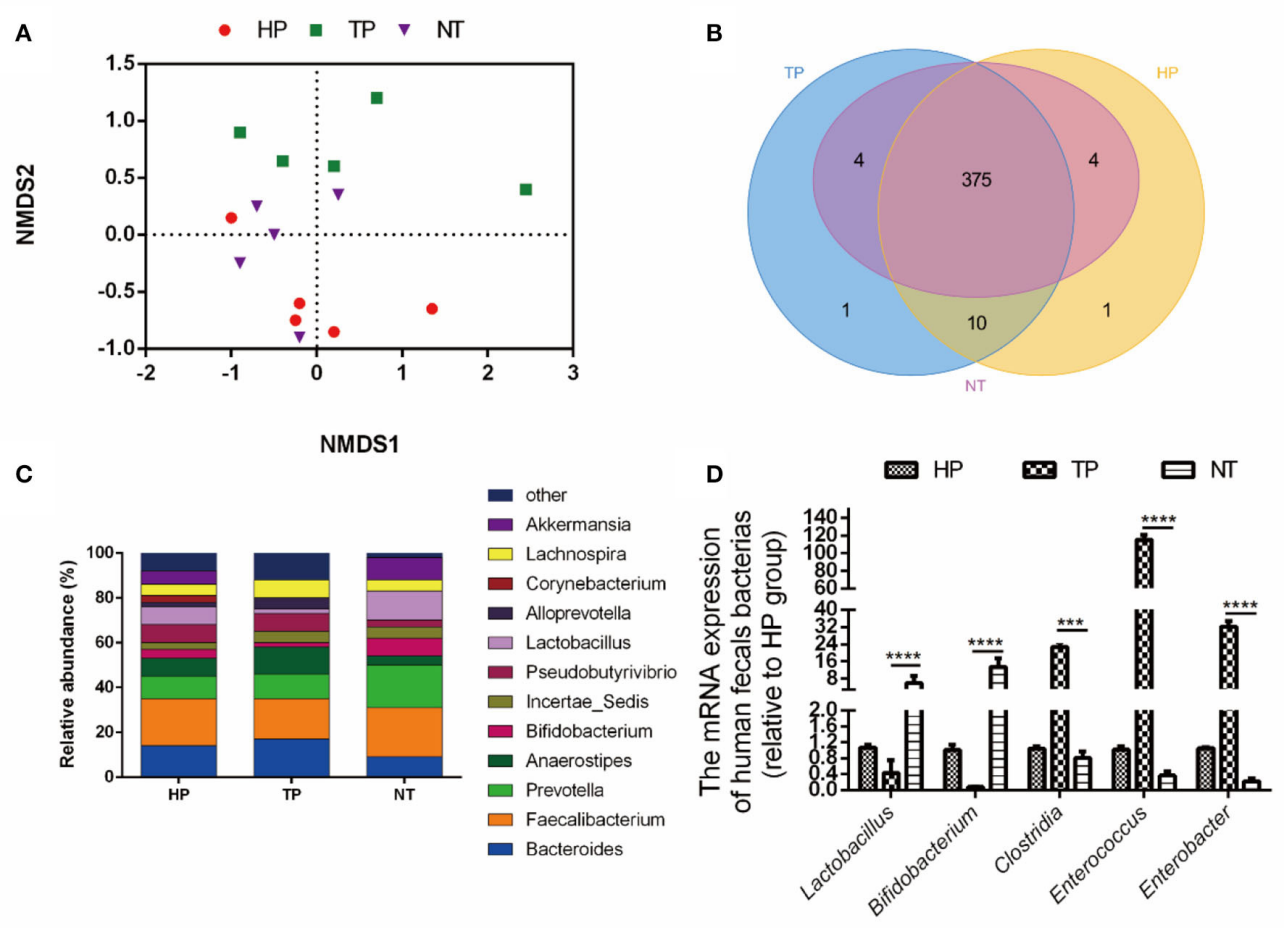
Selection of probiotic strains. **(A)** The non-metric multidimensional scaling (NMDS) analysis of healthy people (HP) from employees of the Jiangxi Cancer Hospital, tumor patients (TP) from the Jiangxi Cancer Hospital, and non-cancer people (NT) (healthy residents at the cancer-free village). **(B)** The Venn diagram of the intestinal microbiota among HP, TP, and NT. **(C)** The relative abundance of the bacteria among groups HP, TP, and NT. **(D)** The relative abundance of bacteria among the HP, TP, and NT groups with quantitative real-time PCR (qRT-PCR). The data are presented as means ± SD, where ***p < 0.001 and **** *p* < 0.0001.

First, a high-throughput sequencing analysis was used to compare the intestinal-microbial diversity among HP, patients with cancer, and cancer-free village residents. The NMDS analysis showed that samples in HP and NT groups clustered together, while they deviated from the samples of patients with tumor (TP group) (Figure 1A). The Venn diagram indicated that 375 common OTUs were observed from HP, TP, and NT groups (Figure 1B), and the decreased relative abundance of probiotics, such as *Lactobacillus* (HP: TP: NT = 8: 2: 13%), *Bifidobacterium*, and *Akkermansia*, was obtained at the genus level (Figure 1C). The results of qRT-PCR further confirmed that the abundance of probiotics, such as *Lactobacillus* and *Bifidobacterium*, in the NT group was higher than that in the HP and TP groups (*p* < 0.001), while the abundance of harmful bacteria, such as *Clostridium, Enterococcus*, and *Enterobacter*, in the NT group was reduced, compared with HP and TP groups (*p* < 0.001; Figure 1D).

Then, the selective MRS medium for *Lactobacillus* was used to selectively isolate *Lactobacilli* and 10 strains, namely *Clostridium tertium* MH282454.1, *Weissellacibaria* KU555931.1, *L. curvatus* LC129556.1, *W. confusa* KC416985.1, *L. plantarum* KJ779102.1, *L. reuteri* KX881777.1, *L. paracasei* MG822869.1, *Enterococcus faecium* KX267939.1, *L. mucosae* FJ751778.1, and *Pediococcuspentosaceus* KJ806297.1, were identified from feces of cancer-free village residents (Supplementary Table 1). Finally, the *L. plantarum* KJ779102.1 was chosen for further study according to the standard of the China Food and Drug Administration. This strain has been stored as a patent bacterium in the Institute of Microbiology, Chinese Academy of Sciences (*L. plantarum* MH-301).

To prepare the probiotic cocktail in clinic trial, *L. plantarum* MH-301, *B. animalis* subsp. *Lactis* LPL-RH, *L. rhamnosusnosus* LGG-18, and *L. acidophilus* were finally chosen for their acid resistance, high resistance to bile salts, strong oxidation resistance, broad spectrum of antibacterial ability, and high cell adhesion (Supplementary Figure 1).

### Probiotic Cocktail Effectively Eliminated the Severity of OM *via* Improving the Immunity of Patients With NPC Receiving CCRT

**Figure 2:**
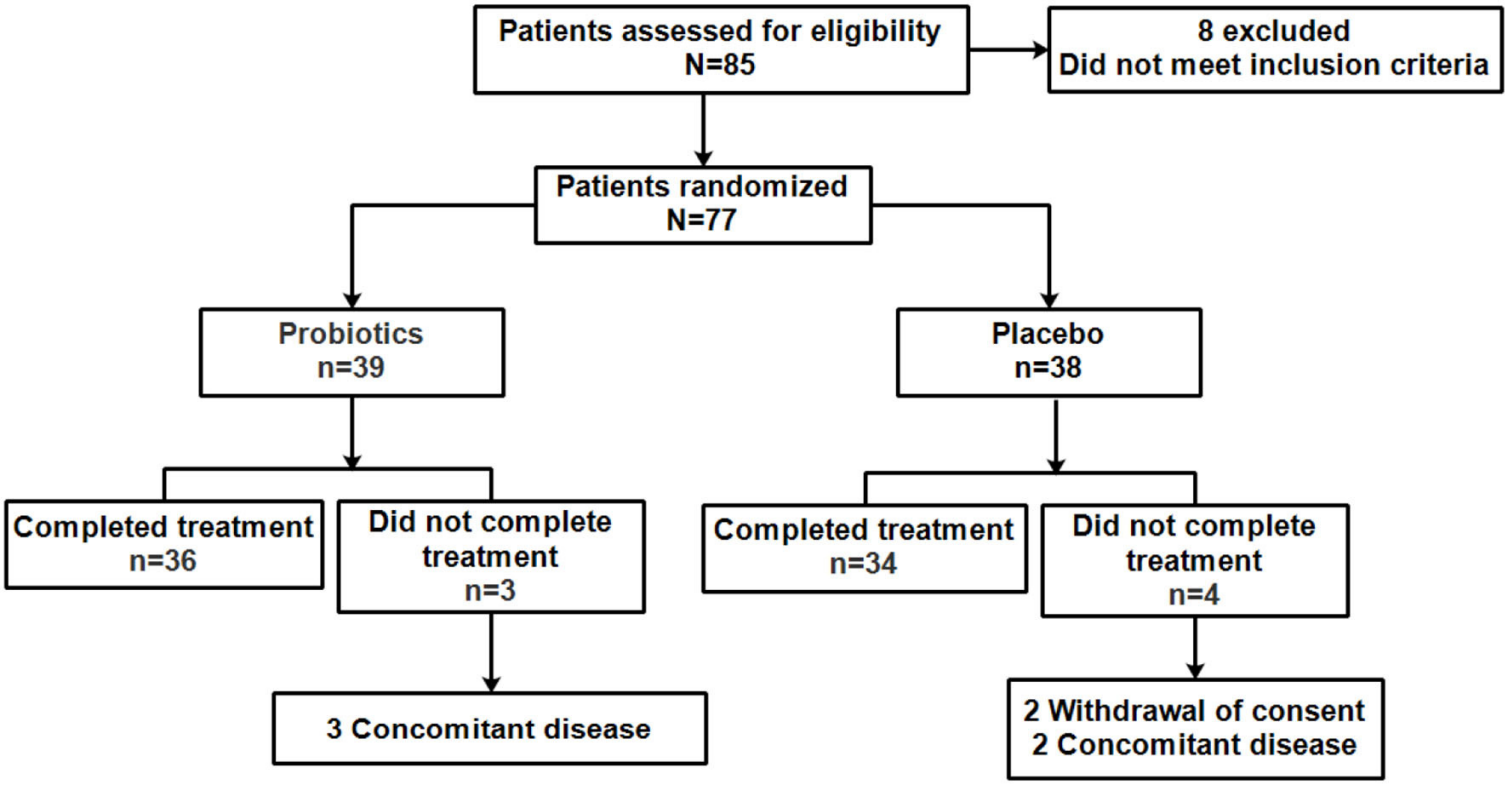
Patients included in the study.

A total of 85 patients were assessed for eligibility evaluation, and eight patients were excluded for their failure to meet the inclusion criteria. The remaining 77 patients were randomly assigned to the probiotics (39 patients) or the placebo group (38 patients) in the ratio of 1:1 patients, three patients in the probiotics group were further excluded due to complication, and four patients in the placebo group were excluded for complication (two patients) or withdrawal of consent (two patients). In the end, 34 patients were finally designated as the placebo group, and 36 patients were finally designated as the probiotic group (Figure 2). There was no marked difference of baseline characteristics in patients between the placebo group and the probiotic group, and the details of gender, age, tumor stage, and node stage of patients who completed the treatment were summarized in Supplementary Table 2.

**Figure 3:**
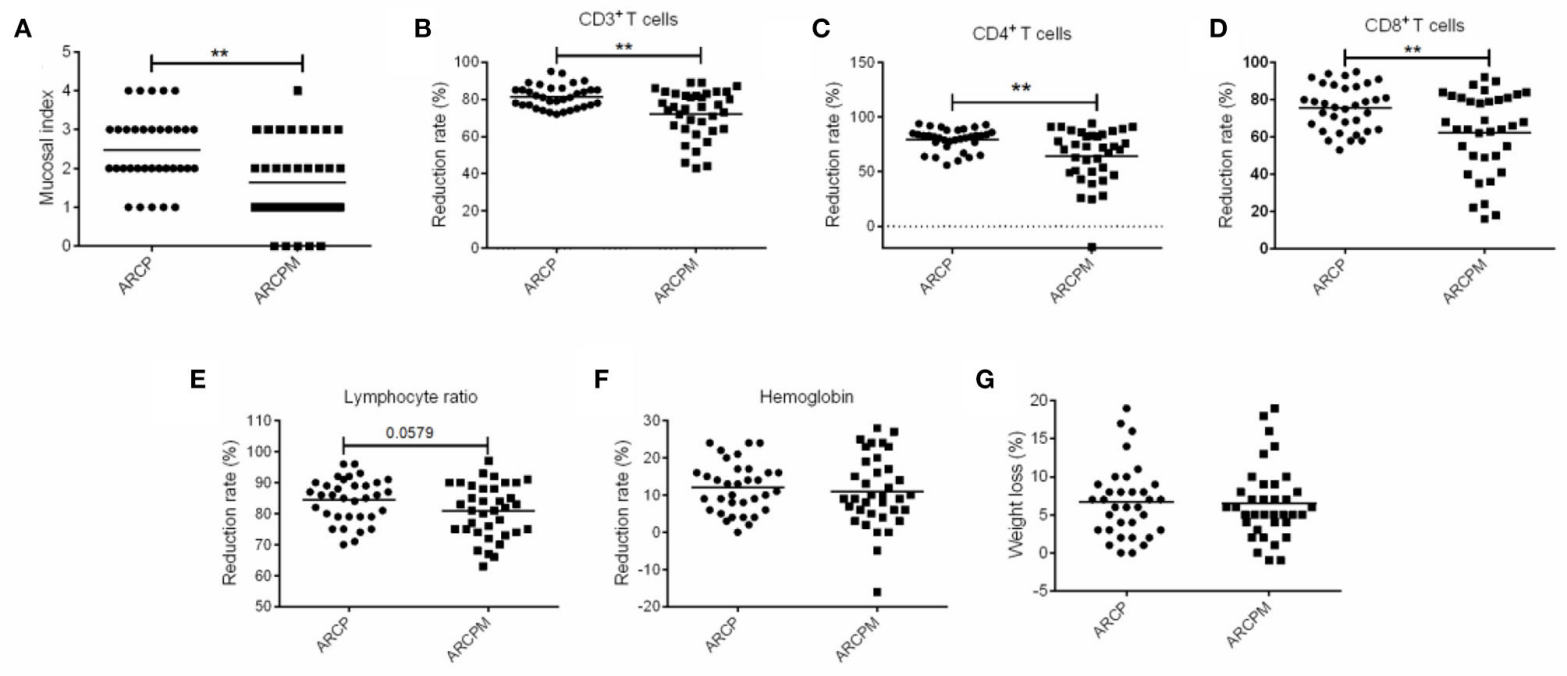
The combination of probiotics reduced oral mucositis (OM) by improving the immunity of patients with nasopharyngeal cancer (NPC). **(A)** Mucosal index. **(B)** Reduction rate of CD3^+^T cells. **(C)** Reduction rate of CD4^+^T cells. **(D)** Reduction rate of CD8^+^T cells. **(E)** Reduction rate of lymphocyte. **(F)** Reduction rate of hemoglobin. **(G)** Weight loss. The data are presented as means ± SD, where **p < 0.01.

As shown in Figure 3A, probiotic cocktail significantly reduced the severity of OM in patients with NPC who underwent CCRT. The rate of incidence of 0, 1, 2, 3, and 4 grades of OM were 0, 14.7, 38.2, 32.4, and 14.7%, respectively, in the ARCP group, while they were 13.9, 36.1, 25, 22.2, and 2.8%, respectively, in the ARCPM group (*p* < 0.01). Moreover, probiotic cocktail significantly attenuated the negative impact of CCRT on immunity. Oral administration of probiotic cocktail greatly enhanced the reduction rate of CD3^+^ T cells (75.5 vs. 81%, *p* < 0.01), CD4^+^ T cells (64.53%vs. 79.53%, *p* < 0.01), and CD8^+^ T cells (75.59 vs. 62.36%, *p* < 0.01) compared to patients in the ARCP group (Figures 3B–D). No significant differences of reduction rate of lymphocyte (80.81 vs. 84.44%, p > 0.05), hemoglobin (10.94 vs. 12%, *p* > 0.05), and body weight (6.53 vs. 6.7%, *p* > 0.05) were observed between the ARCPM and ARCP groups (Figures 3E–G).

### Probiotic Cocktail Altered the Composition of the Intestinal Microbiome in Patients With NPC

**Figure 4:**
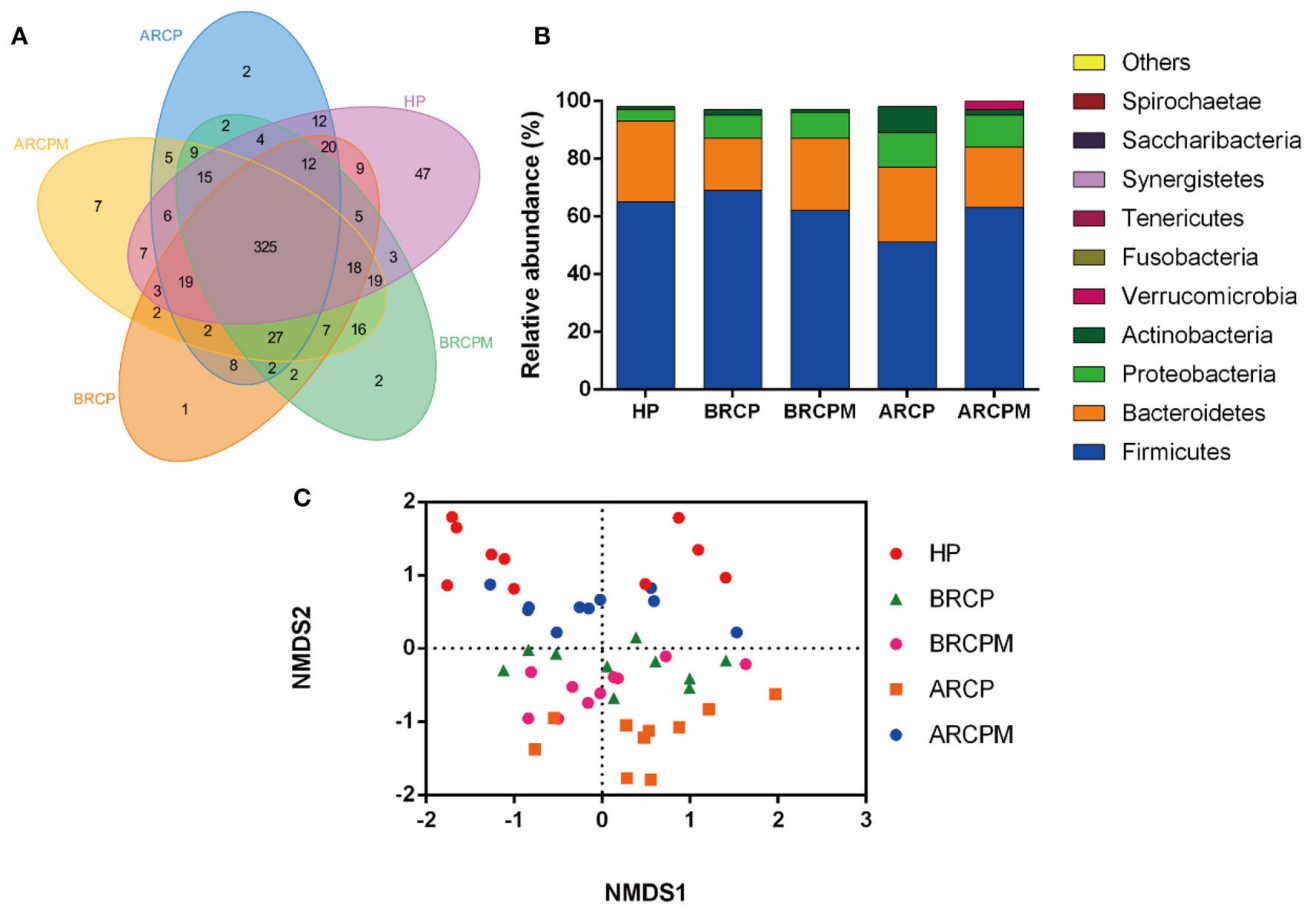
Effects of the combination of probiotics on the composition of bacterial communities in patients with NPC. **(A)** The relative abundance of bacteria among HP from the employees of the Jiangxi Cancer Hospital, before the treatment of radiotherapy plus chemotherapy plus a placebo (BRCP), after treatment with radiotherapy plus chemotherapy plus a placebo (ARCP), before the treatment of radiotherapy plus chemotherapy plus the probiotic combination (BRCPM), and after treatment with radiotherapy plus chemotherapy plus the probiotic combination (ARCPM). **(B)** The Venn diagram of the intestinal microbiota among HP, BRCP, ARCP, BRCPM, and ARCPM groups. **(C)** The NMDS analysis of groups HP, BRCP, ARCP, BRCPM, and ARCPM.

In total, 2,936,897 clean tags and 9,941 OTUs were obtained with an average of 196.4 OTUs in each group (Supplementary Table 3). The Venn diagram reflected the difference of OTUs in all groups. In total, 325 common OTUs were identified in all groups and 47 OTUs were identified specifically in the HP group. Notably, eight OTUs belonged only to the RCP groups (BRCP and ARCP groups), and 16 OTUs were identified exclusively in RCPM groups (BRCPM and ARCPM groups) (Figure 4A).

At the phylum level, the data of the top 10 populations of microorganisms were analyzed. *Firmicute, Bacteroidetes, Proteobacteria*, and *Actinobacteria* were predominated in HP, ARCP, BRCP, ARCPM, and BRCPM groups (*Firmicutes*: 66.03, 52.10, 69.41, 63.30, and 63.10%, respectively; *Bacteroidetes*: 28.02, 27.22, 18.82, 19.01, and 25.30%, respectively; *Proteobacteria:* 4.45, 11.89, 8.942, 11.13, and 9.45%, respectively; and *Actinobacteria*: 1.49, 8.18, 2.20, 3.00, and 1.72%, respectively). The abundance of *Bacteroidetes* and *Actinobacteria* were increased and the abundance of *Firmicutes* was decreased in the ARCP group, but probiotic cocktail enriched the abundance of *Firmicutes* and reduced the abundance of *Bacteroidetes* and *Actinobacteria* to the normal level (Figure 4B).

The NMDS analysis found that BRCPM, ARCPM, BRCP, and HP groups were clustered together, while the ARCP group diverged from other groups and showed a scattered distribution, indicating that the probiotic cocktail restored the gut dysbiosis in patients with NPC who underwent CCRT (Figure 4C).

### Probiotic Cocktail Attenuated Tongue Tissue Inflammation and Pathological Damage in Rats With OM Caused by Radiotherapy and Chemotherapy

**Figure 5:**
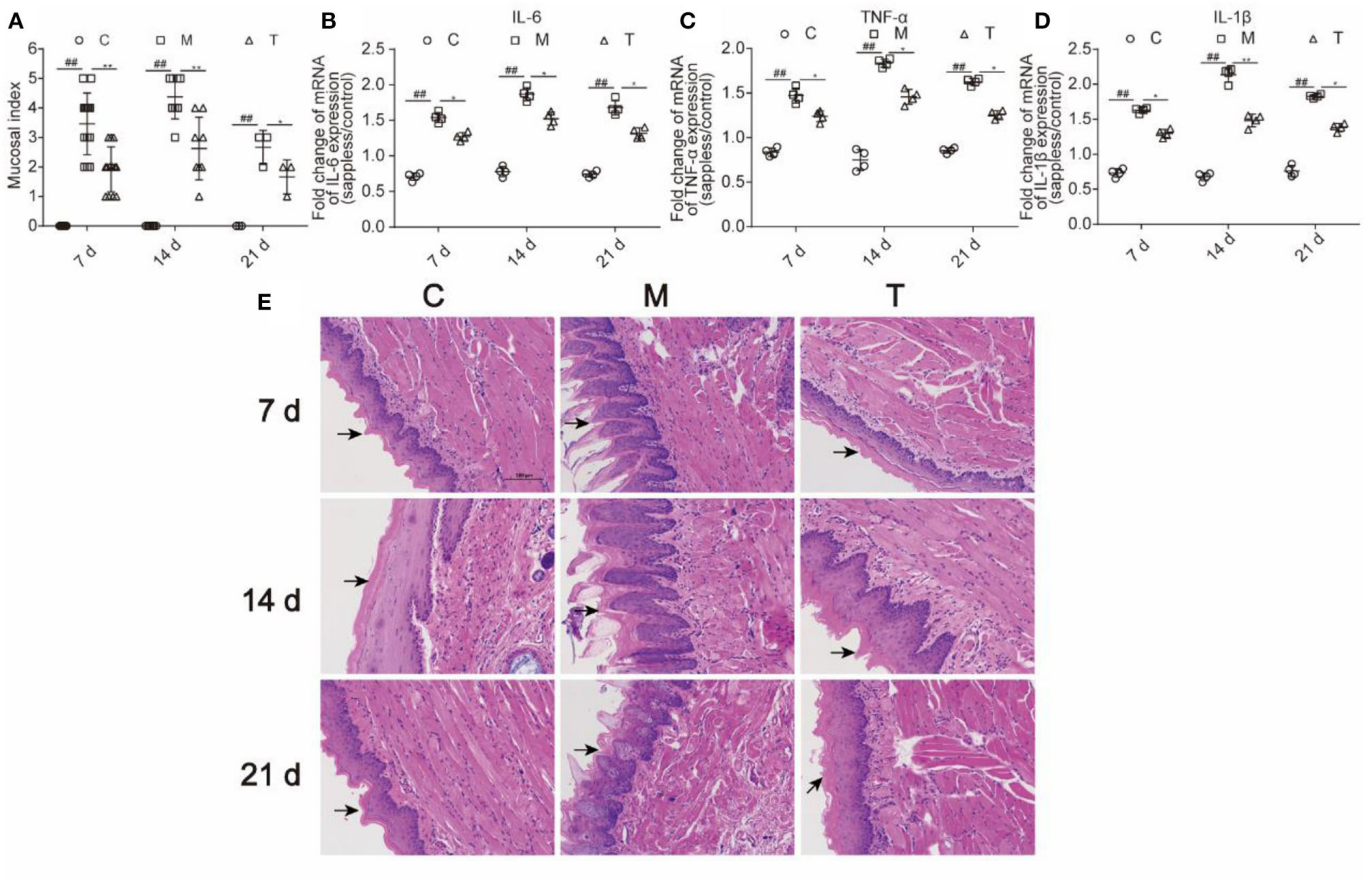
The probiotic cocktail relieved tongue tissue inflammatory response in rats with OM caused by radiotherapy and chemotherapy. **(A)** Mucosal index. **(B)** The expression of IL-6 in the mRNA level. **(C)** The expression level of TNF-α in the mRNA level. **(D)** The expression of IL-1β in the mRNA level. **(E)** H&E staining of the tongue tissue among the C, M, and T groups on the 7th, 14th, and 21st days. The data are presented as means ± SD, where *p < 0.05, **p < 0.01, and ^##^*p* < 0.01.

As shown in Figure 5A, the mucosal index was evaluated on the 7th, 14th, and 21st days. The incidence of grade 3 or over was significantly increased in the M group than those in the C group on the 7th, 14th, and 21st days (76.9 vs. 0%, 100 vs. 0%, and 66.7 vs. 0%; *p* < 0.01; respectively). Otherwise, the incidence of grade 3 or over was significantly decreased in the T group than those in the M group on the 7th, 14th, and 21st day (23.1 vs. 76.9%, 50 vs. 100%, and 0 vs. 66.7%; *p* < 0.01; respectively). It is worth mentioning that the severity of OM was alleviated on the 21st day both in the M and T groups compared with that on the 7th and 14th days.

The hemogram of rats was measured on the 7th, 14th, and 21st days. As shown in Table 1, the concentration of leukocyte was higher in the M group than in the C group on the 7th, 14th, and 21st days (18.39 ± 4.06 vs. 12.38 ± 2.53, *p* < 0.01; 20.65 ± 3.35 vs. 15.06 ± 2.00, *p* < 0.01; 16.63 ± 2.05 vs. 13.70 ± 2.45; *p* < 0.05). However, the concentration of leukocyte was lower in the T group than in the M group (16.32 ± 2.57 vs. 18.39 ± 4.06, *p* < 0.05; 16.80 ± 3.02 vs. 20.65 ± 3.35, *p* < 0.05; 15.90 ± 1.90 vs. 16.63 ± 2.05, *p* < 0.05). This result suggested that probiotic cocktail suppressed peripheral immune response by reducing the level of blood leukocyte in rats that were treated with radiotherapy and chemotherapy.

**Table 1:** Blood routine test of rats.

**Time**	**Group**	**WBC (×10^9^·L^−1^)**	**RBC (×10^12^·L^−1^)**	**HGB (g·L^−1^)**	**PCT (×10^9^·L^−1^)**
7 days	C (*n* = 5)	12.38 ± 2.53	5.62 ± 1.49	121.80 ± 13.47	186.94 ± 16.97
	M (*n* = 5)	18.39 ± 4.06**	5.36 ± 1.40	129.45 ± 11.84	193.14 ± 16.91
	T (*n* = 5)	16.32 ± 2.57^#^	6.35 ± 1.98	122.18 ± 10.24	194.30 ± 20.67
14 days	C (*n* = 5)	15.06 ± 2.00	5.55 ± 1.83	128.08 ± 5.73	194.20 ± 13.48
	M (*n* = 5)	20.65 ± 3.35**	5.33 ± 1.87	127.30 ± 14.12	192.09 ± 13.87
	T (*n* = 5)	16.80 ± 3.02^##^	5.80 ± 1.43	132.15 ± 21.30	199.06 ± 15.02
21 days	C (*n* = 3)	13.70 ± 2.45	5.67 ± 1.79	135.48 ± 26.65	194.87 ± 24.53
	M (*n* = 3)	16.63 ± 2.05*	5.01 ± 2.54	130.33 ± 15.04	195.44 ± 23.62
	T(*n* = 3)	15.90 ± 1.90^#^	5.68 ± 2.89	138.66 ± 34.19	193.64 ± 17.79

The data are presented as means ± SD, where, *p < 0.05, **p < 0.01, ^#^p < 0.05, and ^##^p < 0.01.

To evaluate the inflammation of oral mucosa, expressions of IL-6, IL-1β, and TNF-α in the mRNA level of the tongue tissue were measured (Figures 5B–D). The M group demonstrated significantly higher expression level than the C group in IL-6 (1.54 vs. 0.71, *p* < 0.01; 1.87 vs. 0.78, *p* < 0.01; 1.68 vs. 0.74, *p* < 0.01, respectively), IL-1β (1.63 vs. 0.73, *p* < 0.01; 2.14 vs. 0.68, *p* < 0.001; 1.83 vs. 0.76, *p* < 0.01, respectively), and TNF-α (1.47 vs. 0.84, *p* < 0.01; 1.84 vs. 0.75, *p* < 0.01; 1.63 vs. 0.86, *p* < 0.01, respectively), on the 7th, 14th, and 21st days. The T group showed remarkably lower expression level than the M group in IL-6 (1.27 vs. 1.54, *p* < 0.05; 1.52 vs. 1.87, *p* < 0.05;1.32 vs. 1.68, *p* < 0.05, respectively), IL-1β (1.30 vs. 1.63, *p* < 0.05; 1.48 vs. 2.14, *p* < 0.05; 1.38 vs. 1.83, *p* < 0.05, respectively) and TNF-α (1.24 vs. 1.47, *p* < 0.05; 1.46 vs. 1.84, *p* < 0.05; 1.26 vs. 1.63, *p* < 0.05, respectively) on the 7th, 14th, and 21st days. The result indicated that probiotic cocktail remarkably attenuated oral inflammation in rats that were treated with radiotherapy and chemotherapy.

The histology samples of the tongue tissue of each group were collected on the 7th, 14th, and 21st days. The H&E staining visually reflected the severity of the tongue and its mucosal thickening in the C, M, and T groups. Severe destruction of tongue epithelium, the erosion of the corneum, and the proliferation of basal cells were found in the M group compared with the C group. However, the damage of the tongue tissue in the T group was observed to be lighter than the M group, which indicated that the probiotic cocktail could relieve the inflammatory response. The structural damage of the tongue tissue was observed on the 7th day, deteriorated on the 14th day, and was partly repaired on the 21st day both in the M and T groups (Figure 5E).

These results indicated that the probiotic cocktail inhibited the peripheral immune response, inflammation, and pathological damage, further to alleviate the severity of OM in rats induced by radiotherapy and chemotherapy.

### Probiotic Cocktail Ameliorated Tongue Tissue Apoptosis, Reversed the Upregulation of TLR4/NF-κB, and Improved the Expression of Intestinal Tight Junction in Rats With OM

**Figure 6:**
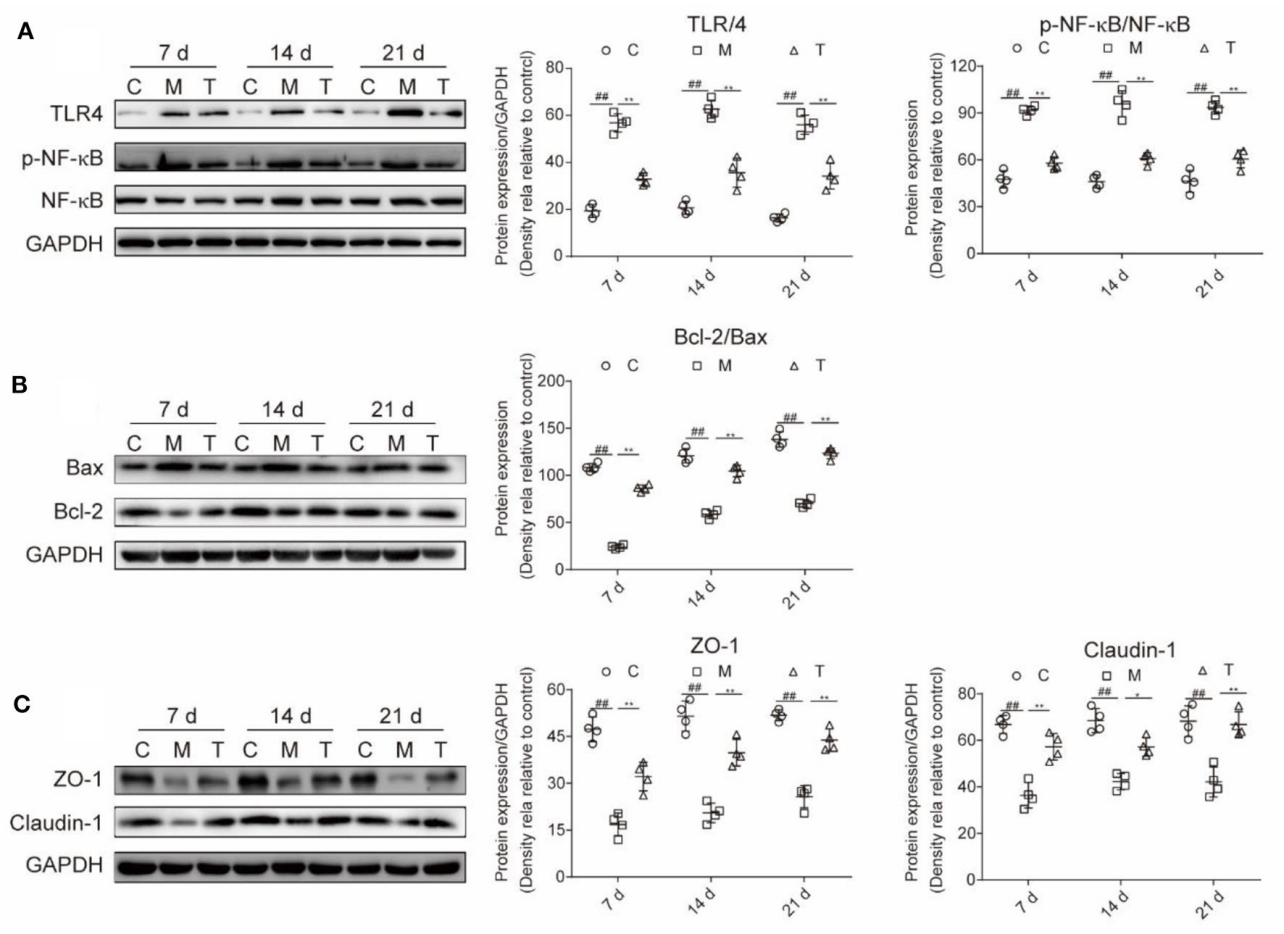
The combination of probiotics ameliorated the upregulation of TLR4/NF-κB, tongue tissue apoptosis and improved the expression of intestinal tight junction (TJ) in rats with OM caused by radiotherapy and chemotherapy. **(A)** Protein expression level of TLR4, P-NF-κB, and NF-κB. **(B)** Protein expression level of apoptosis-associated factors, Bcl-2 and Bax. **(C)** The expression level of intestinal TJ proteins, ZO-1 and claudin-1. The data are presented as means ± SD, where *p < 0.05, **p < 0.01, ^#^*p* < 0.05, and ^##^*P* < 0.01.

Inflammation-related proteins, cell apoptosis, and intestinal TJ proteins were measured in the tongue and colon tissue, respectively (Figure 6). Compared with the C group, a higher expression of TLR4 (7th, 14th, and 21st days) and P-NF-κB/NF-κB (7th, 14th, and 21st days) in the M group were observed (*p* < 0.001). However, the probiotic cocktail markedly downregulated the expression of TLR4 (7th, 14th, and 21st days) and P-NF-κB/NF-κB since the 7th day (*p* < 0.001) in rats with OM. Also, the probiotic cocktail significantly inhibited the apoptosis caused by radiotherapy and chemotherapy *via* reducing the ratio of Bax/Bcl-2 (Figure 6B).

The integrity of the intestinal barrier was measured with intestinal TJ proteins. The results indicated that the expression of ZO-1 (7th, 14th, and 21st days) and Claudin-1 (7th, 14th, and 21st days) were greatly reduced in rats with OM, and the probiotic cocktail markedly restored the expression of ZO-1 (7th, 14th, and 21st days) and Claudin-1 (7th, 14th, and 21st days) to normal levels (Figure 6C).

In summary, the probiotic cocktail attenuates the severity of OM possibly by downregulating the TLR4/NF-κB signaling pathway, reducing cell apoptosis, and downregulating the intestinal TJ proteins.

### Probiotic Cocktail Effectively Restored the Disturbed Microbial Diversity to a Normal Level in Rats With OM

**Figure 7:**
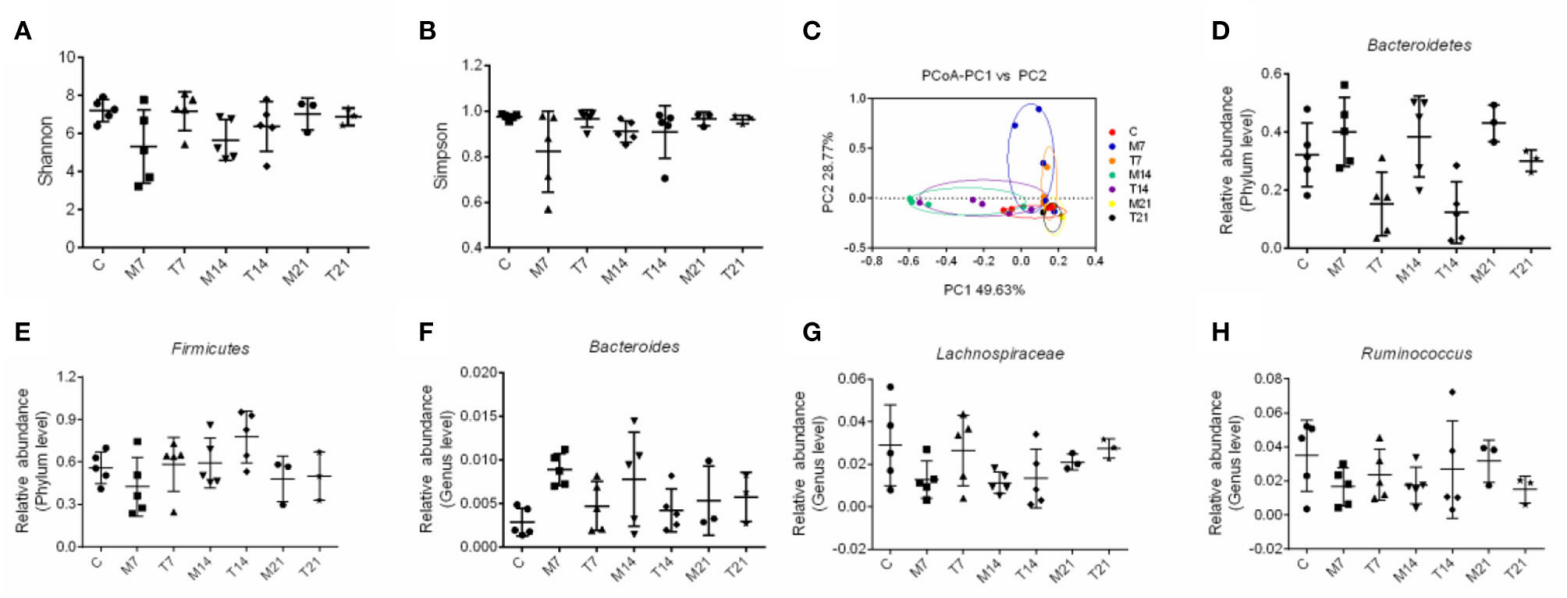
Effects of probiotic cocktail on the composition of bacterial communities in rats with OM caused by radiotherapy and chemotherapy. **(A)** Shannon index of intestinal bacterial communities among C, M, and T groups at days 7, 14, and 21. **(B)** Simpson index of intestinal bacterial communities among C, M, and T groups on the 7th, 14th, and 21st days. **(C)** Principle coordinate analysis (PCoA) of intestinal bacterial communities among C, M, and T groups on the 7th, 14th, and 21st days. **(D)** Relative abundance of *Bacteroidetes* of intestinal bacterial communities at the phylum level among C, M, and T groups on the 7th, 14th, and 21st days. **(E)** Relative abundance of *Firmicutes* of intestinal bacterial communities at the phylum level among C, M, and T groups on the 7th, 14th, and 21st days. **(F)** Relative abundance of *Bacteroidetes* of intestinal bacterial communities at the genus level among C, M, and T groups on the 7th, 14th, and 21st days. **(G)** Relative abundance of *Lachnospiraceae* of intestinal bacterial communities at the genus level among C, M, and T groups on the 7th, 14th, and 21st days. **(H)** Relative abundance of *Ruminococcus* of intestinal bacterial communities at the genus level among the C, M, and T groups on the 7th, 14th, and 21st days.

To test whether the probiotic cocktail could reverse the gut dysbiosis induced by radiotherapy and chemotherapy in rats, we analyzed the composition and community structure of bacteria in feces through 16S rRNA gene sequencing. As illustrated in Figures 7A,B, the probiotic cocktail improved the microbial α-diversity, the Shannon index, and the Simpson index, on the 7th and 14th days, which were decreased in rats with OM, although there was no significant statistical difference. The PCoA analysis showed that fecal microbial populations of animals in the C and T groups clustered together and diverged from that of the rats with OM over the study period, indicating that the probiotic cocktail partially shaped the alterations of the microbial community in rats with OM caused by radiotherapy and chemotherapy (Figure 7C).

The abundance of *Firmicutes* was decreased and the abundance of *Bacteroidetes* showed an increasing trend in the M group on the 7th, 14th, and 21st days. Moreover, anti-inflammation-related bacteria, such as *Lachnospiraceae* and *Ruminococcus*, were reduced and inflammation-related bacteria, such as *Bacteroides*, were enriched in the M group on the 7th, 14th, and 21st days. However, the abundance of *Firmicutes, Lachnospiraceae*, and *Ruminococcus* was increased and the abundance of *Bacteroidetes* and *Bacteroides* was decreased in the T group than that of the M group, though there was no significant statistical difference (Figures 7D–H). These results suggested that the probiotic cocktail shaped gut dysbiosis and prevented inflammation in radiotherapy and chemotherapy-induced rats.

## Discussion

In this study, we investigated the impact of the combination of *L. plantarum* MH-301, *B. animalis* subsp. *Lactis* LPL-RH, *L. rhamnosus* LGG-18, and *L. acidophilus* on OM for the first time. As expected, the results showed that 47.1% of the patients in the ARCP group developed ≥grade 3 OM, whereas OM was only 25% developed in the ARCPM group, and this supported the protective effect of the probiotic cocktail against OM. Consistent with the clinical test, the probiotic cocktail also reduced the severity of grade 3 OM (23.1 vs. 76.9%, 50 vs. 100%, and 0 vs. 66.7%) on the 7th, 14th, and 21st days in radiotherapy and chemotherapy-induced rats. In addition, the probiotic cocktail also improved the immunity of patients with NPC and restored gut dysbiosis to normal both in the patients with NPC and rats with OM induced by radiotherapy and chemotherapy.

Recently, a series of studies have indicated that gut microbiota might modulate the response to immunotherapy in cancer treatment (30, 31). It was found that treating mice with probiotic *L. plantarum* (KC836552.1) significantly reduced tumor volume and activated immune responses, such as enhanced levels of CD8^+^ T and NK cells in patients with cancer (32). Recently, it has become evident that the ability of gut microbiota to regulate immunity in cancer therapy modulates the susceptibility to toxic side effects (8, 33). *Bifidobacterium* ameliorated chemotherapy-induced mucositis *via* promoting the expression of CD4^+^T cell immunity in rats with cancer (34). Moreover, evidence has suggested that probiotics initiate memory in the T and B cells, trigger adaptive immunity, and activate the immune system which may stimulate the production of salivary glycoproteins and antimicrobial peptides, finally protecting the oral mucosa from damage (35). As reported in an earlier study (13), this study also showed that RCPM significantly enhanced the number of T-cells (CD3^+^ T, CD8^+^ T, and CD4^+^ T cells) and decreased the severity of OM in comparison with those of patients with RCP, which further confirmed that probiotics modulate human immune responses to cancer treatment, eventually reducing the related side effects (36). Besides, it seemed that the effect of this probiotic cocktail on patients with OM and NPC was better than the combination of the previous probiotics (*B. longum, L. lactis, and E. faecium)*.

We used an animal model to verify the assumption that the probiotic cocktail might have beneficial effects on OM induced by radiotherapy and chemotherapy and to make a primary exploration of the mechanisms. In our study, we observed the severity of the oral damage caused by radiotherapy and chemotherapy in rats. The pathogenesis of oral inflammation induced by chemotherapy and irradiation is complex. It had been proposed that radiotherapy and chemotherapy cause DNA and non-DNA damage to the epithelium of cells, tissues, and blood vessels (37). It could also cause reactive oxygen species, followed by the activation of TLR4/NF-κB pathway, which could promote the production of pro-inflammation factors (TNF-α, IL-1β, and IL-6), and accelerate apoptosis (Bax/Bcl-2), and ultimately aggravated tissue damage and lead to bacterial, viral, and fungal infections (38, 39). TNF-α, IL-6, IL-1β, and cell apoptosis played a critical role in the development of mucositis (40). Moreover, evidence has indicated that the IL-6 level positively correlated with the severity of mucositis both in radiation-induced OM mice and in the head and neck of patients with cancer who underwent radiotherapy or radiochemotherapy (41, 42). The results demonstrated that probiotic cocktail administration diminished the upregulation of TLR4/NF-κB and elevated the levels of pro-inflammatory cytokines and cell apoptosis caused by chemotherapy and irradiation.

The intestinal epithelial barrier prevents the entry of exterior antigens from the gut lumen into the host, which may exacerbate both local and systemic immune responses (43). The front line of this barrier is composed of epithelial cells and apical junctional complexes encompassing TJ proteins between the adjacent epithelial cells (44). Previous studies had found that CCRT treatment for cancer also aggravated the dysfunction of the intestinal barrier and caused peripheral immune activation and inflammation (45–47). As expected, the results also showed that TJ proteins (ZO-1 and Claudin-1) were reduced and neutrophils were increased in the M group. Besides, the expression of proteins forming TJ was also influenced by gut microbiota. Probiotics have been shown to increase the expression of TJ protein and restore intestinal permeability, eventually suppressing peripheral neutrophils (48, 49). In line with this study, ZO-1 and Claudin-1 were found to increase in the T group, suggesting that probiotics prevented system-immune activation and inflammation, including an increase in TNF-α, IL-1β, and IL-6 in the oral cavity, which eventually could ameliorate OM.

Radiotherapy and chemotherapy also change intestinal microbiota, which leads to altered colonic epithelial cell homeostasis, impaired barrier function, and increased susceptibility to OM (46, 50, 51). The NMDS analysis was conducted, and our clinical experiments suggested that CCRT had obviously disturbed the diversity of gut microbiota, and the samples from the ARCP group were scattered far from the samples from the HP group, whereas the administration of the mixture of probiotics markedly restored the microbial diversity in the ARCP group than that of the HP and ARCPM groups. Similar to the results of the clinical trial, our rat model also indicated that the bacterial communities had recovered back to normal after being treated with the probiotic cocktail for 21 days with the PCoA. This suggested that the probiotic cocktail had significantly reduced the side effects of CCRT by sustaining the bacterial homeostasis of the intestines.

The gut microbiome of T group rats also reflected enriched species-richness as well as a significant shift in the overall microbial diversity at the phylum and genera level compared to the M group. Recent studies have also revealed that some disadvantage of bacterial strains were increased, while the bacterial strains which were beneficial for health were reduced in patients with cancer who underwent CCRT (52, 53). Accordingly, compared to control, we observed a lower relative abundance of *Firmicutes* and a higher relative abundance of *Bacteroidetes* at the phylum level both in patients with NPC and in mice with OM. We also found that a higher abundance of *Actinobacillus* was observed in patients with NPC than those in HP. In addition, higher *Bacteroides*, lower *Lachnospiraceae*, and lower *Ruminococcus* at the genus level were observed in mice with OM. Based on the previous study, *Actinobacillus* were usually found to be both an oral symbiotic and an opportunistic pathogen, which were associated with the pathogenesis of meningitis, sinusitis, pleural empyema, and bronchopneumonia (54, 55). Besides, *Actinobacillus* might seriously affect the homeostasis of oropharyngeal microorganisms, and was one of the susceptible factors for patients with NPC who had severe mucositis (55). *Bacteroides* have been observed to be a prominent feature in patients with inflammatory bowel disease (56) and are associated with mucus degradation and a pro-inflammatory phenotype (57). Other bacteria in the *Firmicutes* phylum, such as *Lachnospiraceae* and *Ruminococcaceae*, demonstrated an anti-inflammatory process by reducing pro-inflammatory cytokines (IL-12 and IFN-γ) and increasing the anti-inflammatory cytokines (IL-10) (58). In addition to the anti-inflammatory activity, *Lachnospiraceae* and *Ruminococcaceae* were also considered to be associated with the butyrate-producing process (59). Butyrate was suggested to be important in ameliorating mucosal inflammation and maintaining the intestinal barrier (60). It is important to emphasize that the intestinal ecosystem is partially important to maintain human health. Specific changes, such as the decrease of *Firmicutes* and the increase of *Bacteroidetes* in this ecosystem, may contribute to the development of inflammatory-related disease (61). Taken together, the increase in the abundance of inflammation-related *Bacteroidetes* and *Actinobacillus* and a decrease in the abundance of anti-inflammation-related *Firmicutes* in patients with NPC who were treated with CCRT may have affected the severity of OM to some extent.

Probiotics, such as *L. lactis* and *B. longum*, were expected to be useful for intestinal inflammation and OM (62, 63). A recent study also demonstrated that a mix of *Bacillus subtilis* (2.9 × 10^8^ CFU/g), *B. bifidum* (2.0 × 10^8^ CFU/g), *E. faecium* (2.1 × 10^8^CFU/g), and *L. acidophilus* (1.0 × 10^8^ CFU/g) reduced the histological severity of intestinal mucositis and OM in rats treated with chemotherapy (64). Probiotic-based treatments have proven to be beneficial for chemotherapy- or radiotherapy-induced mucositis, possibly by regulating the microbiome and inhibiting the pro-inflammatory cytokines (65, 66). In the same way, *B. bifidum* G9-1(BBG9-1) eliminates 5-FU-induced mucositis by inhibiting secondary inflammation through a reduction in the abundance of *Bacteroidetes* and the corresponding increase in the abundance of *Firmicute*s (67). In addition, *Lactobacillus* significantly reversed the chemotherapy- or radiation-disturbed composition of *Firmicutes* and *Bacteroidetes*, thereby reducing pro-inflammatory reactions and mucositis (46, 68, 69). Thus, the changed abundance of *Firmicute*s and *Bacteroidetes* induced by radiotherapy and chemotherapy were associated with intestinal inflammation, to further induce or increase the incidence of OM, and the probiotic cocktail decreased the severity of OM by regulating the homeostasis of intestinal bacteria.

There are some strengths in our study. First, we isolated *L. plantarum* from free village residents. Secondly, according to human clinical and rat trials, probiotic combinations, namely *L. plantarum, B. animalis* subsp. *Lactis* LPL-RH, *L. rhamnosusnosus* LGG-18, and *L. acidophilus*, might reduce the severity of OM in patients with NPC who were treated with radiotherapy and chemotherapy. Finally, our results indicated that the probiotic cocktail might alleviate the severity of OM in patients with NPC who were treated with radiotherapy and chemotherapy by regulating gut microbiota dysbiosis and enhancing immunity. There are some limitations in this study. One disadvantage is that the number of patients with NPC was not large enough and more patients with NPC are needed in the future to confirm the results; another limitation is the need for fecal microbiota transplantation to further identify the role of gut microbiota on the effectiveness of probiotic cocktail on OM of patients with NP.

These results indicated that the probiotic cocktail could significantly reduce the severity of OM in patients with NPC, which might be related to improving the immunity of patients with NPC and regulating gut microbiota homeostasis. The results of the probiotic cocktail on rats with OM induced by radiotherapy and chemotherapy further confirmed that the probiotic cocktail could ameliorate the severity of OM by modulating the gut dysbiosis related to inflammatory responses.

## Data Availability Statement

The datasets presented in this study can be found in online repositories. The names of the repository/repositories and accession number(s) can be found in the article/Supplementary Material.

## Ethics Statement

This study was approved by the local clinical research Ethics Committee and was conducted following the Helsinki declaration (No. 2017ky023). All the patients gave their informed consent before the trail. The patients/participants provided their written informed consent to participate in this study. The animal study was reviewed and approved by Animal care and procedures were under the National Institutes of Health Guidelines for the Care and Use of Laboratory Animals, and all experiments were approved by the Ethical Committee of the of Nanchang University.

## Author Contributions

TC, HX, and HW conceived and designed the study. CX, TC, and CJ did the data processing and wrote the first draft of the paper. TC, CX, CJ, JW, WL, HH, JL, LF, HW, and HX checked and revised the first draft of the paper. All authors contributed to the article and approved the submitted version.

## Conflict of Interest

The authors declare that the research was conducted in the absence of any commercial or financial relationships that could be construed as a potential conflict of interest.
