# Supplementary material for: A Phase II Randomized Clinical Trial and Mechanistic Studies Using Improved Probiotics to Prevent Oral Mucositis Induced by Concurrent Radiotherapy and Chemotherapy in Nasopharyngeal Carcinoma
Source: Front Immunol. 2021 Mar 24;12:618150. doi: 10.3389/fimmu.2021.618150 (PMC8024544; doi:10.3389/fimmu.2021.618150)
Supplement: Supplementary file 1 [file Table_1.docx]

TABLE S1 Strains isolated from human feces in cancer-free village

| Strains No. | Closest relatives | Similarity (%) | GeneBank No. |
| --- | --- | --- | --- |
| 1 | *Clostridium tertium* | 96 | MH282454.1 |
| 2 | *Weissella cibaria* | 98 | KU555931.1 |
| 3 | *Lactobacillus curvatus* | 98 | LC129556.1 |
| 4 | *Weissella confusa* | 98 | KC416985.1 |
| 5 | *Lactobacillus plantarum* | 99 | KJ779102.1 |
| 6 | *Lactobacillus reuteri* | 98 | KX881777.1 |
| 7 | *Lactobacillus paracasei* | 98 | MG822869.1 |
| 8 | *Lactobacillus casei* | 97 | JN560859.1 |
| 9 | *Enterococcus faecium* | 98 | KX267939.1 |
| 10 | *Lactobacillus mucosae* | 98 | FJ751778.1 |
| 11 | *Pediococcus pentosaceus* | 99 | KJ806297.1 |
