# Supplementary material for: A Phase II Randomized Clinical Trial and Mechanistic Studies Using Improved Probiotics to Prevent Oral Mucositis Induced by Concurrent Radiotherapy and Chemotherapy in Nasopharyngeal Carcinoma
Source: Front Immunol. 2021 Mar 24;12:618150. doi: 10.3389/fimmu.2021.618150 (PMC8024544; doi:10.3389/fimmu.2021.618150)
Supplement: Supplementary file 2 [file Table_2.docx]

TABLE S2 Baseline patient demographics and characteristics

| Variable | RCP group (N=34) | RCPM group (N=36) | P value |
| --- | --- | --- | --- |
| Percentage of total enrollment, No. (%) | 34(48.57) | 36 (51.43) | / |
| Male : female, n:n (%:%) | 11:23 (32.00:68.00) | 11:15 (31.00:69.00) | 0.872 |
| Age, mean (SD), y | 51.70[±](http://www.baidu.com/link?url=pNuyEeISk8YpQJxPLZAC2SnfjooCbiBnjpFlCJYGXeRdeCq1Xpc_1JNCe9ER4qEoHRHXet9KpIhnH2gHoolZiJCgD0_0pYn0Ya8qLKnDxAq)11.21 | 52.61[±](http://www.baidu.com/link?url=pNuyEeISk8YpQJxPLZAC2SnfjooCbiBnjpFlCJYGXeRdeCq1Xpc_1JNCe9ER4qEoHRHXet9KpIhnH2gHoolZiJCgD0_0pYn0Ya8qLKnDxAq)10.56 | 0.729 |
| Tumor stage, No. (%) |  |  |  |
| T1 | 0 (0) | 1 (3.00) | 0.905 |
| T2 | 2 (6.00) | 3 (8.00) |  |
| T3 | 13 (38.00) | 14 (39.00) |  |
| T4 | 19 (56.00) | 18 (50.00) |  |
| Node stage, No. (%) |  |  |  |
| N0 | 8 (24.00) | 9 (25.00) | 0.724 |
| N1 | 14 (41.00) | 17 (47.00) |  |
| N2 | 9 (26.00) | 9 (25.00) |  |
| N3 | 3(9.00) | 1(3) |  |
