# Supplementary material for: A Phase II Randomized Clinical Trial and Mechanistic Studies Using Improved Probiotics to Prevent Oral Mucositis Induced by Concurrent Radiotherapy and Chemotherapy in Nasopharyngeal Carcinoma
Source: Front Immunol. 2021 Mar 24;12:618150. doi: 10.3389/fimmu.2021.618150 (PMC8024544; doi:10.3389/fimmu.2021.618150)
Supplement: Supplementary file 3 [file Table_3.docx]

TABLE S3 Number of total tag, taxon tag, and OTUs in groups H, BRCP, ARCP, BRCPM and ARCPM by high‑throughput sequencing.

| Sample ID | total tag | taxon tag | OUT num |
| --- | --- | --- | --- |
| HP1 | 59138 | 59075 | 324 |
| HP2 | 51864 | 51804 | 314 |
| HP3 | 50400 | 50376 | 266 |
| HP4 | 50187 | 50144 | 327 |
| HP5 | 54798 | 54497 | 340 |
| HP6 | 70882 | 68728 | 167 |
| HP7 | 64467 | 62175 | 170 |
| HP8 | 72965 | 71882 | 219 |
| HP9 | 60255 | 57563 | 175 |
| HP10 | 63484 | 61032 | 134 |
| BRCP1 | 67298 | 65771 | 261 |
| BRCP2 | 72043 | 70709 | 246 |
| BRCP3 | 53484 | 52734 | 210 |
| BRCP4 | 60767 | 59605 | 211 |
| BRCP5 | 71475 | 70110 | 181 |
| BRCP6 | 60299 | 59186 | 188 |
| BRCP7 | 55566 | 54650 | 200 |
| BRCP8 | 53173 | 52121 | 232 |
| BRCP9 | 68536 | 67971 | 128 |
| BRCP10 | 55636 | 54802 | 167 |
| ARCP1 | 58380 | 58367 | 169 |
| ARCP2 | 62003 | 61995 | 185 |
| ARCP3 | 62195 | 62182 | 132 |
| ARCP4 | 60122 | 60018 | 173 |
| ARCP5 | 62314 | 61214 | 217 |
| ARCP6 | 63553 | 60560 | 184 |
| ARCP7 | 66767 | 63085 | 193 |
| ARCP8 | 52303 | 51241 | 269 |
| ARCP9 | 52075 | 51824 | 162 |
| ARCP10 | 62576 | 61747 | 110 |
| ARCPM1 | 71489 | 69218 | 211 |
| ARCPM2 | 48462 | 47222 | 169 |
| ARCPM3 | 43278 | 43111 | 208 |
| ARCPM4 | 51154 | 50301 | 140 |
| ARCPM5 | 69147 | 68216 | 119 |
| ARCPM6 | 54484 | 52080 | 212 |
| ARCPM7 | 60248 | 58069 | 246 |
| ARCPM8 | 61542 | 59275 | 180 |
| ARCPM9 | 71348 | 66991 | 152 |
| ARCPM10 | 56385 | 55177 | 266 |
| BRCPM1 | 61437 | 56899 | 165 |
| BRCPM2 | 53192 | 52576 | 129 |
| BRCPM3 | 70080 | 67564 | 183 |
| BRCPM4 | 54886 | 52460 | 132 |
| BRCPM5 | 50174 | 49926 | 149 |
| BRCPM6 | 54048 | 51973 | 214 |
| BRCPM7 | 70909 | 68710 | 183 |
| BRCPM8 | 59964 | 58554 | 202 |
| BRCPM9 | 62566 | 60616 | 195 |
| BRCPM10 | 62374 | 60791 | 232 |
| Total | 3006172 | 2936897 | 9941 |
| Average | 60123.44 | 58737.94 | 198.82 |
