# Supplementary material for: A Phase II Randomized Clinical Trial and Mechanistic Studies Using Improved Probiotics to Prevent Oral Mucositis Induced by Concurrent Radiotherapy and Chemotherapy in Nasopharyngeal Carcinoma
Source: Front Immunol. 2021 Mar 24;12:618150. doi: 10.3389/fimmu.2021.618150 (PMC8024544; doi:10.3389/fimmu.2021.618150)

FIGURE S1 The prebiotic evaluation of *L. Plantarum* MH-301, *B. Animalis* subsp.*lactis* LPL-RH, *L. Rhamnosus* LGG-18 and *L. Acidophilus*.

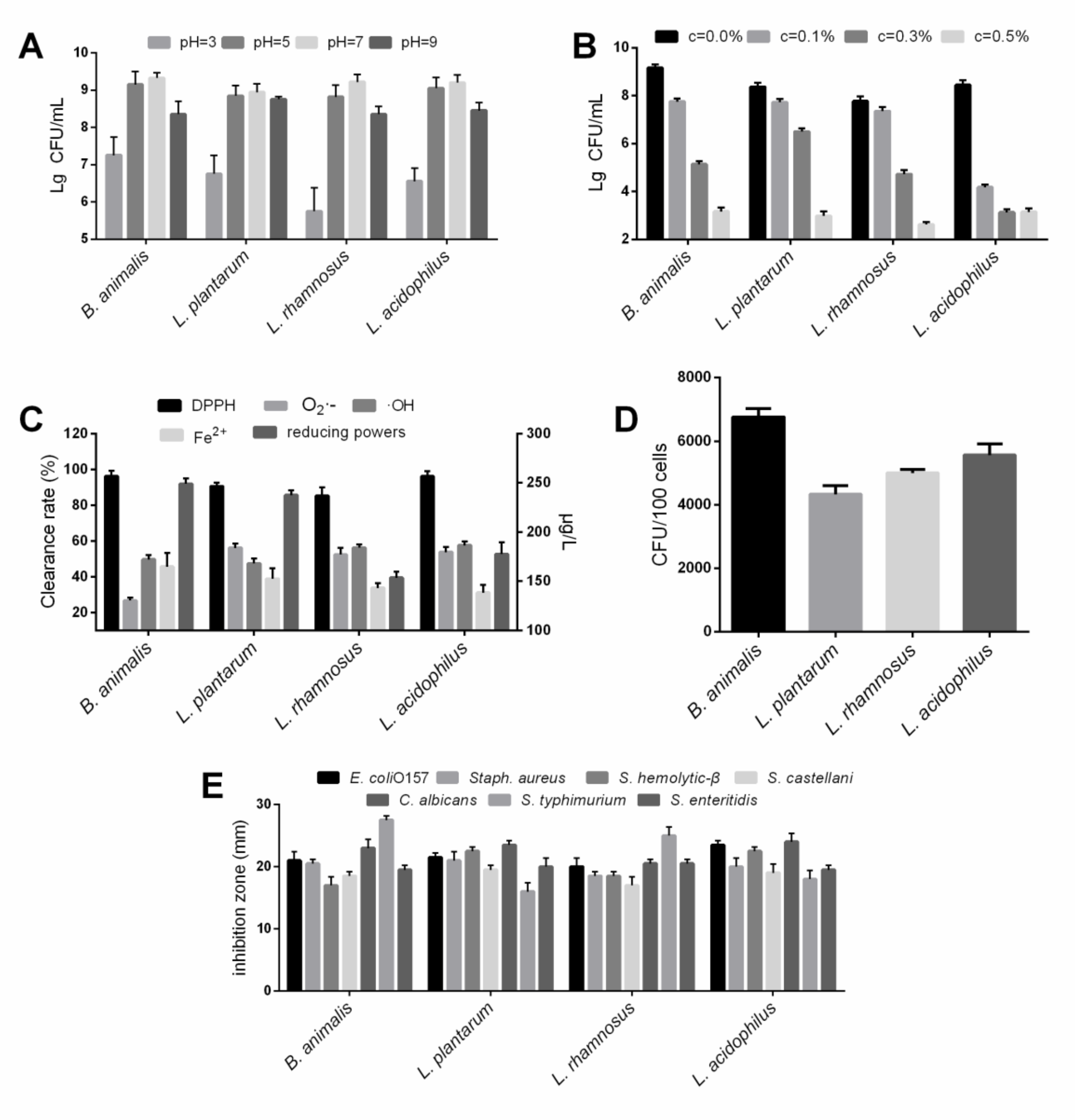

Supplement: Supplementary file 4 [file Image_1.pdf]
