# Supplementary figures and images for: A Phase II Randomized Clinical Trial and Mechanistic Studies Using Improved Probiotics to Prevent Oral Mucositis Induced by Concurrent Radiotherapy and Chemotherapy in Nasopharyngeal Carcinoma
Source: Front Immunol. 2021 Mar 24;12:618150. doi: 10.3389/fimmu.2021.618150 (PMC8024544; doi:10.3389/fimmu.2021.618150)

**FIGURE S2 Representative pictures to relieve OM in rats with the combination of probiotics.**

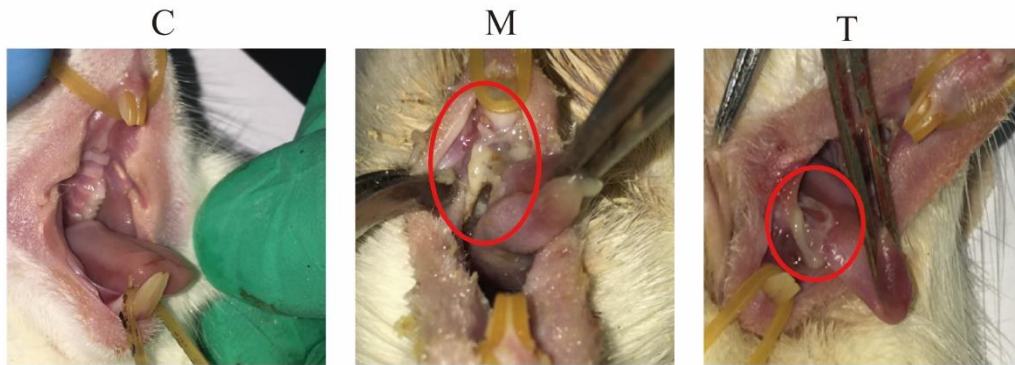

Supplement: Supplementary file 5 [file Image_2.pdf]
